# Screening First Degree Relatives of Persons with Primary Open Angle Glaucoma in India

**DOI:** 10.5005/jp-journals-10008-1172

**Published:** 2015-01-15

**Authors:** Sharmila Rajendrababu, Nidhi Gupta, B Vijayakumar, R Kumaragurupari, SR Krishnadas

**Affiliations:** Senior Glaucoma Consultant, Department of Glaucoma, Aravind Eye Hospital, Madurai Tamil Nadu, India; Mediacal Officer, Aravind Eye Care System, Madurai, Tamil Nadu, India; Biostatistcian, Aravind Eye Care System, Madurai, Tamil Nadu, India; Librarian, Aravind Eye Care System, Madurai, Tamil Nadu, India; Professor, Department of Glaucoma Services, Aravind Eye Care System Madurai, Tamil Nadu, India

**Keywords:** Primary open angle glaucoma, Family glaucoma, Family history, Heredity history, Heredity, Risk factor, Targeted factor, Targeted screening, First degree relatives.

## Abstract

**Purpose:** To report the results of screening first degree relatives of persons identified with primary open angle glaucoma in a tertiary eye hospital glaucoma services.

**Design:** A cross-sectional study of first degree relatives of persons with primary open angle glaucoma.

**Materials and methods:** First degree relatives of patients identified with primary open angle glaucoma were invited to participate in a screening evaluation in the base hospital to detect glaucoma. All participating individuals had comprehensive eye examination including vision screening, refraction, slit-lamp biomicroscopy, applanation tonometry, gonioscopy, frequency doubling peri-metry and dilated fundus examination. Persons with definite and suspected glaucoma were subject to full threshold automated perimetry.

**Results:** A 514 first degree relatives of 346 persons with primary open angle glaucoma, of 4972 individuals who were invited to participate attended the screening examination (Response Rate 7%). Fifty-five percent of those who attended were males and mean age of participants was 56.8 years. Sixty-eight relatives (13.3% of those screened) were detected to have definite glaucoma. Sixty percent of those detected with definite glaucoma were siblings. Fifteen percent of siblings, 4% of off-springs and 20% of parents who attended the screening examination had definite open angle glaucoma.

**Conclusion:** Prevalence of open angle glaucoma amongst first degree relatives of persons with glaucoma is higher than in the general population as reported in previous studies. Significant barriers, however, exist in the uptake of eye care services among relatives of persons known to have primary open angle glaucoma.

**How to cite this article:** Rajendrababu S, Gupta N, Vijayakumar B, Kumaragurupari R, Krishnadas SR. Screening First Degree Relatives of Persons with Primary Open Angle Glaucoma in India. J Curr Glaucoma Pract 2014;8(3):107-112.

## INTRODUCTION

Glaucoma is the second most common cause of blindness^1^ and is an important cause of irreversible visual loss in India and the world. The World Health Organization has estimated that 12.3% of the 37 million people blind worldwide is attributed to glaucoma.^1^ It has been predicted that prevalence of glaucoma will exponentially increase to 79.6 million with an estimated 11 million persons bilaterally blind from glaucoma by the year 2020.^2^ India has the largest population of persons with blindness globally. Various population based studies have estimated the prevalence of glaucoma in India ranges from 1.62 to 3.51%.^3^ Approximately 11.2 million persons in India aged 40 years and older are estimated to have glaucoma. Primary open angle glaucoma is the most predominant form of glaucoma estimated to affect 6.48 million persons.^3^ Quigley and Broman^2^ in their landmark publication had projected that the most detectable change in glaucoma worldwide in the current decade will be its significant increase in India. Most of those with the disease are undetected and there exist major challenges in detecting and treating those with this chronic asymptomatic disease. Population based surveys indicate that glaucoma remains undiagnosed in 90% of those affected,^3,4^ in the developing world. There is a need to improve screening and case detection, and to develop methods that can be applied worldwide and in less developed countries in an attempt to prevent needless blindness.

Glaucoma screening is a critical tool for reducing incidence of blindness and vision loss from a predominantly asymptomatic disease. Detection of glaucoma in earlier stage of the disease by effective screening is likely to substantially reduce morbidity, improve quality of life and reduce healthcare expenditure related to glaucoma. Prevention of vision loss from glaucoma significantly depend on availability of sensitive and specific tests for detecting the disease earlier in populations at risk. Population based screening, however, have had limited effectiveness in glaucoma detection primarily because it is a difficult disease to diagnose in a definitive manner given low sensitivity of IOP measurement as a screening tool and high variability in the appearance of optic nerve heads. Relatively low prevalence of glaucoma in the general population also renders its screening less cost effective except for specific subgroups at higher risk.^5^ Screening may be useful in populations with a higher prevalence of glaucoma such as the elderly,^6,7^ African American ancestry,^8^ Hispanics^9,10^ and those with a family history of glaucoma,^11^ since the positive predictive value of available screening tests is greatly enhanced in populations at higher risk of glaucoma.

The increased risk of glaucoma in family members of persons with POAG has been well-recognized and several studies have reported on screening of relatives of individuals with open angle glaucoma. Epidemiologic data from the Baltimore Eye Survey^12^ confirm that family history of POAG is an important risk factor. Cross sectional studies seem to suggest close to 50% of all glaucomas to be familial and a positive family history confers threefold increase in risk of developing open angle glaucoma.^12,13^ The risk ratio of developing primary open angle glaucoma in persons with a positive family history has been estimated to be 9.2 and population studies evaluating the role of family history of glaucoma have observed an increased prevalence of open angle glaucoma among the first degree relatives of persons with POAG.^11,14^ Barbados eye study has suggested that persons most likely to have POAG are older men with family history of glaucoma.^15^ Examination of family members of those diagnosed with POAG could be an effective means to identify those at greater risk of glaucoma facilitating earlier detection and treatment.^16^ A longitudinal screening of siblings of a cohort of white patients with POAG, the Nottingham Family Glaucoma Screening Study^17^ reported an increased risk of developing glaucoma in siblings with increasing age. The present study reports screening of first degree relatives of persons with open angle glaucoma in a South Indian population, where utilization of eye care services has been reported to be less than optimal. It is hypothesized that targeted screening of families of individuals diagnosed with primary open angle glaucoma in these populations will facilitate earlier diagnosis of glaucoma in persons at a higher risk of developing the disease. Appropriate treatment and follow-up of such individuals is likely to prevent needless blindness.

## MATERIALS AND METHODS

The current glaucoma family study recruited probands of primary open angle glaucoma diagnosed at the glaucoma services, Aravind Eye Hospital at Madurai between June 2007 and July 2009 and their families who were invited to participate in the screening program after obtaining an informed consent from the probands. The family study was approved by the Institutional Review Board and Ethics Committee of the Aravind Eye Hospitals.

All Consecutive persons with newly diagnosed primary open angle glaucoma in the study period were explained about the glaucoma family screening initiative by a study coordinator and requested to provide the name, age, sex, nature of relationship and mailing address of their first degree relatives. An informed consent was obtained from all probands to contact their first degree relatives to invite them to participate in screening to detect glaucoma. The mailing sent to them included:

1. A letter describing the screening program and inviting participation.
2. A brochure highlighting the familial association of glaucoma, need to detect the disease early and treat to prevent vision impairment and unnecessary blindness.

The screening was held on second sunday of every month at the premises of the Aravind Eye Hospital and was offered free of cost to all participants to encourage acceptance for the screening initiative. Those who could not attend on the appointed day were encouraged to report on any week day at their convenience to report to the glaucoma services of the hospital to facilitate screening.

All the available family members were invited to undergo a complete ocular examination which included visual acuity and refraction, slit-lamp biomicroscopy of the anterior segment, applanation tonometry, central corneal thickness, gonioscopy, frequency doubling perimetry and a dilated fundus examination with a 90D Indirect lens. Families of probands with glaucoma other than the primary open angle glaucoma such as primary angle closure and exfoliative glaucomas were excluded from this study. Individuals with definitive glaucoma based on typical glaucomatous optic nerve damage, ocular hypertension, suspicious appearing optic nerve heads or nerve fiber defects and those with abnormal frequency doubling perimetry examination were subject to visual field testing by standard full threshold automated static perimetry [Swedish Interactive Threshold Algorithm (SITA) 24-2 on the Humphrey Visual Field Analyzer, Zeiss-Humphrey Systems, Dublin, CA].

Slit-lamp examination was performed on all participants, and gross external, corneal, iris, lenticular and vitreous abnormalities were noted. The anterior chamber depth was inspected and pupillary responses carefully noted. Intraocular pressures were measured by Goldman applanation tonometry three times in each eye and median IOP in either eye was observed. Gonioscopy was performed in either eye in all individuals with Goldman two mirror gonioscopy lens and were graded by Shaffer’s classification. Individuals with narrow anterior chamber angles on gonioscopic evaluation were further excluded from the study and were offered treatment as appropriate. Direct and indirect ophthalmoscopy with a 90D lens was performed after dilatation of pupils and the vertical cup/ disk ratio, neural rim thinning, notching of optic nerve head, nerve fiber hemorrhages, and retinal nerve fiber layer abnormalities were noted. Optic disk photographs were not taken. In addition to a detailed clinical ocular examination, all individuals were also subject to frequency doubling perimetry by N-30 threshold strategy (FDT perimeter from Humphrey-Zeiss Systems, Dublin, CA with frequency doubling technology from Welch Allyn, NY). All clinical examination including tonometry, fundus examination and gonioscopy were performed by an experienced glaucoma specialist. Visual acuity and refraction, CCT and perimetry were performed by trained paramedical personnel.

Primary open angle glaucoma was confirmed based on the presence of the following criteria:

1. Uncharacteristic, open angles documented on gonio-scopy.
2. An optic nerve head cup to disk ratio greater than 0.6 with typical features of notching of neuroretinal rim, excavation, thinning or sloping of neural rim in the superior or inferior sector of the optic nerve head in at least one eye.
3. Visual field abnormalities matching optic nerve head changes on SITA 24-2 with pattern standard deviation abnormal at 5% level or less; with a cluster of at least three abnormal points in the nasal, paracentral or arcuate region of the visual field with at least one point at p < 1%. Borderline or abnormal Glaucoma hemifield test on automated perimetry alone without matching disk changes were not considered abnormal. Persons with typical optic nerve head changes suggestive of glaucomatous damage without typical, matching visual field changes had repeat visual field examinations. The investigators believed a diagnosis based on characteristic optic disk changes and corresponding visual field changes ensured a high sensitivity and specificity for detection of glaucomatous damage.

Individuals were classified to have ocular hypertension if median IOP ≥ 21 mm Hg repeatedly on two independent visit, without characteristic optic disk changes or visual field defects typical of glaucoma. Patients with large cup to disk ratio with neural rim slope with no evidence of characteristic neural rim notch or thinning, median IOP < 21 mm Hg on two repeated visits and no visual field defects characteristic of glaucoma were termed glaucoma suspects. We included for analysis only the family members who were subject to a complete ophthalmologic evaluation by the study team to confirm or exclude glaucoma. If probands informed any of their family members had glaucoma but had not reported for the screening conducted by the investigators, they were not included for analysis.

## RESULTS

A total of 860 individuals (473 males/387 females) including 346 probands and their 514 first degree relatives were examined in this study. Of the 4972 persons detected with primary open angle glaucoma whose families were invited to participate in the family screening initiative in this tertiary hospital during the study period, there were only 514 first degree relatives from 346 families who attended the screening with a response rate of 7%. All individuals were of Tamil ethnic origin and natives of the south Indian state of Tamil Nadu. The relatives of POAG probands ranged in age from 21 to 86 years (Mean 56.85 ± years) with 55.5% being males. Of the 514 first degree relatives who had responded to screening request, 79 (15%) were parents, 266 (52%) were siblings and 169 (33%) were offsprings of the probands. Siblings were more likely to have responded to the family glaucoma screening request than parents or offsprings. Sixty-eight first degree relatives (13.3%) were observed to have definite primary open angle glaucoma based on optic nerve head and visual field criteria, and an additional 28 (5.5%) persons were categorized as either ocular hypertensives (elevated IOP > 21 mm Hg without demonstrable optic nerve head or visual field changes) or glaucoma suspects based on suspicious, but not unequivocal optic nerve head appearance without matching visual field defects. Persons with definite glaucoma were distributed as follows: 41 siblings (60%), 20 parents (29%) and 7 (10%) children. Definite glaucoma was observed in 15% of siblings, 4% of off-springs and 20% of the parents of probands who had responded to the invitation for screening.

## DISCUSSION

Prior studies^12,18,19^ have established that primary open angle glaucoma is more likely to affect persons with a family history of the disease and a positive family history has been assumed to be associated with a significant risk of glaucoma. A proportion of POAG is known to have genetic origin as demonstrated by family pedigrees conforming to dominant and recessive modes of Mendelian inheritance.^20,21^ A positive family history has also been reported in 13 to 25% of patients with POAG in the studies from the west,^22,23^ which is 7 to 15 times higher than the prevalence rates of 1.6 to 3.5% observed in population based studies on glaucoma. Proportion of POAG patients classified to be hereditary^21-24^ has ranged from 13 to 50%. Baltimore Eye Study^12^ and Barbados Eye Studies^31^ had reported family history of glaucoma to be a significant risk for primary open angle glaucoma, although these studies had relied on patient reporting of family history of glaucoma, rather than direct examination of the relatives. A self reported status or diagnosis of glaucoma or family history of glaucoma is likely to underestimate the true prevalence of the disease, which can only be ascertained by a detailed ocular examination of all the members of families of those known to have open angle glaucoma. Several studies^22,25-27^ have examined relatives of persons with glaucoma and have found significantly elevated prevalence of the disease (2.8 to 13.5% being affected) compared with controls or that is expected from the prevalence of the disease in the general population (0.52.0%). Several ocular parameters significantly affecting POAG, such as IOP, cup to disk ratio and facility of aqueous outflow are genetically determined^28^ further supporting familial occurrence of this asymptomatic disease. The Barbados family study of glaucoma^32^ was the first to investigate transmission pattern of open angle glaucoma in a predominantly black population and reported 10% open angle glaucoma and 13% probable open angle glaucoma in the relatives of probands who had a comprehensive eye examination. To the best knowledge of the authors, the impact of family history as risk factor for open angle glaucoma or prevalence of glaucoma in the relatives of persons diagnosed with the disease has not been studied previously in an Indian population cohort.

Sixty-eight of the 514 first degree relatives (13.3%) from families of persons with glaucoma enrolled in this study fulfilled the diagnostic criteria of primary open angle glaucoma. The prevalence of familial primary open angle glaucoma in the current study is much lesser than that reported in similar studies in other populations. Nguyen et al^14^ had reported definite glaucoma in nearly a third (30.2%) of the first degree relatives examined, while Vegini et al^29^ had diagnosed open angle glaucoma in 17% of the first degree relatives of probands examined. The higher proportion of affected family members in Nguyen’s study compared with similar reports could be due to inclusion of families with two or more persons with definitive primary open angle glaucoma, which is more representative of individuals with heritable forms of open angle glaucoma. Investigators in the present study had invited the families of all consecutive patients diagnosed with primary open angle glaucoma in the two year period from June 2007 to July 2009 and only a fraction of the families (514 relatives of 346 probands, 7% of the newly detected POAG individuals) had responded to an invitation to participate in the family screening, despite explaining the higher risk of glaucoma in relatives of persons known to have the disease.

Moreover, not all the first degree relatives of the families that have responded to our invitation had attended the screening. This could have resulted in under estimation of the prevalence of glaucoma in relatives of persons with primary open angle glaucoma in the current study. Study coordinator did not send reminders or make any further attempt to contact the nonresponders. Authors believe consistent efforts to trace these members could have significantly enhanced the detection rates of glaucoma in the family members. The study had not been designed to ascertain the disease status of the relatives who had not attended the screening. It is possible that eliciting such history of the disease from nonresponders, who perhaps have had examination elsewhere, could have resulted in increased detection of glaucoma among the first degree relatives. In a cross-sectional survey of biologically related persons of OAG probands, 80% had an eye examination within a year preceding the questionnaire and 18% had been diagnosed and treated for glaucoma^16^ which is higher than that reported in the present study. It is possible that awareness of the increased risk of glaucoma in those related to affected persons is significantly higher in the developed world which could partially explain the higher prevalence due to enhanced detection in these population. A major short coming of the study by Okeke^16^ et al has been that the status of glaucoma in the interviewed candidates had been affirmed through a questionnaire and not by a direct examination, and the reported disease status in relatives is self reported and subject to inaccuracies in diagnosis. There are currently no studies which have attempted to assess knowledge and awareness of glaucoma among the relatives of persons diagnosed with glaucoma in India. Population based assessment of awareness and knowledge^30^ about glaucoma in both rural and urban populations in India, however, has been reported to be very poor. Efforts to educate the public of the increased risk of open angle glaucoma in relatives of affected persons could significantly influence the health seeking behavior in the developing communities leading to increased participation in screening and enhanced detection of glaucoma. Targeted screening and education of first degree relatives of individuals with primary open angle glaucoma is likely to enhance those detected with glaucoma in the community. Apart from inadequate knowledge of risk of glaucoma among the first degree relatives and unwillingness to get screened, significant variations in prevalence of the disease in the cohort studied are also likely to reflect true differences in inheritability of glaucoma in our population, which requires further study.

In our study, a significant proportion of those with definite glaucoma (60%) were siblings of probands with primary open angle glaucoma. A higher prevalence of glaucoma in siblings of those known to have open angle glaucoma has been reported earlier. The Rotterdam eye study^11^ had reported 10% prevalence of glaucoma in siblings as against 1.1% in off-springs of persons with POAG. Nguyen et al^14^ had also reported that siblings among the first degree relatives have the highest risk of glaucoma. The Nottingham family screening study^17^ observed prevalence in siblings to be comparable (11.8%) and had prospectively followed up a cohort of siblings of persons with POAG for a period of 7 years and reported a cumulative incidence of 1% per year of POAG. The incidence of open angle glaucoma in this cohort was higher than the incidence of POAG reported in the general population.^13,33,34^ The higher prevalence of glaucoma in siblings of persons with POAG is explained by the close genetic make-up and constitution and similar environment shared by the siblings during childhood. Although close to two thirds of persons with definite glaucoma in the first degree relatives in the current study were siblings, it is difficult to conclude from our data if siblings were at higher risk of glaucoma than other first degree relatives in our population since we have been unable to examine all the first degree relatives in the families who responded for screening. Most of the first degree relatives who participated in our screening were likely to be siblings (52%) and definite glaucoma was detected in 15% of the siblings who were screened. Fifteen percent of the participants from the probands’ families were parents and definite glaucoma was detected in 20% of those examined. Siblings of probands rather than parents were more likely to have attended the screening, representing an element of bias. The mean age of probands diagnosed with open angle glaucoma in the population was 60.8 years. It is likely that the parents of many of the probands were either deceased or unable to attend the screening owing to physical infirmity or poor health. Smaller sample of parents who had been screened and their higher age could have resulted in a higher prevalence of glaucoma in this group rather than in siblings. Further, a longitudinal follow-up of all the siblings is required to determine the incidence of glaucoma in this group of individuals at risk since all available studies have confirmed increasing age to be a significant risk factor for glaucoma.

A major shortcoming of the present study has been the poor response rate (7%) from the families of persons with POAG to subject to a screening examination. Several factors have possibly contributed to the poor response from the first degree relatives of the probands with POAG. Families in South Tamil Nadu have increasingly become nuclear with migration of the children and siblings and hence it was difficult for the study coordinator to contact and ensure the presence of all the first degree relatives. A few of the persons detected with definite primary open angle glaucoma declined to reveal the disease status to their siblings and hence their families have not been available for screening and examination. It is possible that some of the family members were unable to attend the screening evaluation offered by the investigators but had been examined elsewhere. Information on their disease status have not been made available to the investigators. Availability of such information is likely to have resulted in observation of an increased prevalence of POAG in these families. About 15% of the mails to the families of probands also had returned owing to incorrect mailing address. It is possible that poor knowledge and awareness among the relatives that glaucoma could be hereditary and that all relatives require periodic screening has also contributed to the reluctance of the eligible family members to subject to screening. The authors or the study coordinator made no attempts over the mail to assess the awareness or knowledge of the family members of glaucoma or its risk factors. Prior studies have reported poor knowledge and awareness^31^ of glaucoma among similar populations in South India. Future studies should be designed to assess the awareness of glaucoma among the families of persons with open angle glaucoma and also understand the barriers to uptake of eye care services by first degree relatives of individuals diagnosed with glaucoma.

In summary, we assessed the prevalence of glaucoma in first degree relatives of persons diagnosed with POAG in a South Indian population who had attended a comprehensive screening examination and observed a higher prevalence of definite glaucoma in this cohort than in a general population. Targeting first degree relatives of persons with primary open angle glaucoma may offer a relatively inexpensive way of detecting open angle glaucoma and in the identification of suspects at risk of glaucoma who may be advocated closer monitoring to detect glaucoma early. Glaucoma screening in families of persons with open angle glaucoma is likely to be yet another effective step in reducing the burden of needless blindness from glaucoma in developing societies like India and elsewhere where less than 10% of those with glaucoma in the community have been detected and treated and utilization of eye care services has been low. It has been suggested that 1 in 8 persons with open angle glaucoma has a living relative with undetected glaucoma.^35^ Eye care providers have to be more proactive in recommending periodical examinations for the family members of individuals with POAG. Screening first degree relatives may be a cost-effective method to find at least a proportion of about 90% of open angle glaucoma who are presently undiagnosed in the developing communities. Significant barriers, however, seem to exist in getting the first degree relatives for glaucoma screening which requires further study and need to be addressed in our efforts to prevent avoidable blindness due to glaucoma.
